# The reciprocal interaction between fluoride release of glass ionomers and acid production of *Streptococcus mutans* biofilm

**DOI:** 10.1080/20002297.2022.2055267

**Published:** 2022-03-23

**Authors:** Jian-Na Cai, Hyeon-Mi Choi, Kwang-Yeob Song, Jae-Gyu Jeon

**Affiliations:** a Department of Oral Biology, Binzhou Medical University, Yantai, Shandong, China; b Department of Dentistry, Presbyterian Medical Center, Jeonju, Republic of Korea; c Research Institute of Clinical Medicine of Jeonbuk National University-Biomedical Research Institute of Jeonbuk National University Hospital, Jeonju, Republic of Korea; d Department of Prosthodontics, School of Dentistry, Jeonbuk National University, Jeonju, Republic of Korea; e Department of Preventive Dentistry, School of Dentistry, BK21 Plus Program, Jeonbuk National University, Jeonju, Republic of Korea

**Keywords:** Secondary caries, dental biofilm, restorative materials, fluoride, relationship

## Abstract

**Objectives:**

The aim of this study was to demonstrate the mode of action of glass ionomers (G-Is) against cariogenic biofilms in the slow fluoride release phase by analyzing the reciprocal interaction between fluoride release from G-Is and acid production of *Streptococcus mutans* biofilm.

**Methods:**

G-Is discs in the slow fluoride release phase were prepared and 51 h-old *S. mutans* biofilms were formed on these discs. The interrelationship between the acid production of the biofilm and the fluoride release of the G-Is discs was investigated by analyzing both factors simultaneously during the biofilm formation period. The composition of the 51 h-old biofilms was then examined using microbiological, biochemical, and confocal laser scanning microscopic methods.

**Results:**

Acid production by the cariogenic biofilm, particularly at < pH 5, promotes G-Is fluoride release. Conversely, G-Is fluoride release inhibits the acid production of the cariogenic biofilm. This reciprocal interaction results in the reduction of virulence such as extracellular polysaccharides formation and cariogenic biofilm bio-mass, which may reduce the potential of secondary caries development around G-Is.

**Conclusions:**

These results suggest that G-Is may play a role in preventing the development of secondary caries during the slow fluoride release phase.

## Introduction

Secondary caries is the most common cause of dental restoration replacement [1]. Similar to dental caries, secondary caries results from the interaction of specific bacteria with constituents of the diet within cariogenic biofilms [2]. Caries-related bacteria utilize dietary carbohydrates and produce acid, resulting in the demineralization of the adjacent hard tooth tissues [3]. Furthermore, *Streptococcus mutans*, a primary cariogenic bacterium, can synthesize extracellular polysaccharides (EPSs) from sucrose. These EPSs contribute to the physical integrity and stability of biofilm matrixes, increasing biofilm virulence [4]. Therefore, restorative materials that can effectively reduce the virulence of cariogenic bacteria need to be systematically investigated.

Fluoride played a major role in the dramatic decline in dental caries from the 1950s to the 1980s as it promotes remineralization and slows down demineralization of dental tissue [5]. To control secondary caries, fluoride-releasing materials such as glass ionomers (G-Is) and resin composites have been developed and widely used for decades [6]. Fluoride released from these materials, especially G-Is, was shown to be effective on adjacent hard tooth tissues [7]. However, despite the fact that secondary caries is a direct result from cariogenic biofilm formation, few studies have investigated the way fluoride releasing materials exert their influence on cariogenic biofilms [8–10].

It is known that fluoride release in these materials occurs in a biphasic pattern [11]. G-Is release the largest amount of fluoride during the first day of oral application (rapid fluoride release phase) and show a continuous decrease in fluoride release thereafter (slow fluoride release phase) [12]. This suggests that, if G-Is anti-caries activity is dependent on the amount of its fluoride release as determined in the biphasic pattern, G-Is are predicted to lose their anti-caries activity within a relatively short period of time. However, we recently reported that G-Is fluoride release does not rapidly decrease in the presence of cariogenic biofilms even over long periods of time. Furthermore, we found that G-Is were effective against cariogenic biofilm formation even during their slow fluoride release phase [8]. These results clearly entail that i) factors caused by cariogenic biofilms have a significant impact on G-Is fluoride release and that ii) these factors, in turn, are also affected by G-Is fluoride release.

Previous studies have identified pH as the most important factor that influences G-Is fluoride release (a much greater amount of fluoride ions is released at acidic and cariogenic pH levels) [13]. However, the mutual influence between G-Is fluoride release during the slow release phase and cariogenic biofilm acid production has not been clearly described. As such, the mode of action of G-Is against cariogenic biofilms and the subsequent development of secondary caries are not yet clear.

In this study, we investigated the interaction between fluoride release from G-Is (during the slow release phase) and cariogenic biofilm acid production, and revealed the mode of action of the materials against cariogenic biofilms. The findings we report may offer a new perspective in the area of G-Is’ anti-caries activity.

## Materials and methods

### G-Is disc preparation and biofilm formation on HA or G-Is discs

The materials used in this study are listed in the Supplementary Table. Four commercially available G-Is restorative materials were selected: GC Fuji Filling LC (GC), Ketac Fil Plus Aplicap (Ketac), Riva self-cure HV (Riva), and Unifil Flow (Unifil). These materials were used to prepare disc-shaped specimens (12 mm in diameter and 1.2 mm in thickness) according to previous studies [8]. The G-Is discs were then immersed in 50 mM potassium phosphate (PP) buffer (pH 7) for 240 h to induce the slow fluoride release phase (Supplementary Figure S1). These discs were autoclaved and stored for future experimentation. Hydroxyapatite discs (HA) (12 mm diameter and 1.2 mm thickness; Clarkson Chromatography Products, Inc., South Williamsport, PA) were used as controls in this study.

*S. mutans* UA159 (ATCC 700610) biofilms were formed on the prepared G-Is or HA discs and placed in a vertical position in 24-well plates [14]. Initially, these 24-well plates were filled with 1% sucrose (w/v) ultra-filtered (10 kDa molecular-weight cut-off) tryptone-yeast extract broth with *S. mutans* UA159 (2–5 × 10^6^ colony forming units (CFUs)/ml) and the biofilms were allowed to grow undisturbed for 22 h. From this time point (22 h) onward, the culture medium was changed twice daily (9 am, 6 pm) until it reached 51 h old.

### Experimental scheme

The experimental scheme for this study is detailed in Supplementary Figure S2. To investigate the mode of action of G-Is in the slow fluoride release phase against cariogenic biofilms, the following hypotheses were tested: 1) changes in the pH of the environment may affect the fluoride release of G-Is without cariogenic biofilms, 2) changes in the pH of the environment may affect the fluoride release of G-Is with cariogenic biofilms, 3) changes in fluoride release of G-Is may influence acid production of cariogenic biofilm, 4) in G-Is with cariogenic biofilm growth, fluoride release of G-Is and acid production of biofilm influence each other simultaneously. 5) as a result of this interrelationship between G-Is fluoride release and biofilm acid production, the final composition of cariogenic biofilms can be influenced. After testing the above hypotheses, the mode of action of G-Is against cariogenic biofilms during the slow fluoride release phase was outlined.

### Influence of pH changes on fluoride release of G-Is without biofilms

HA or G-Is discs in the slow fluoride release phase were transferred to a 12-well plate (6 ml salt solution and 2 discs/well) containing a salt solution of varying pH (50 mM KCl plus 1 mM MgCl_2_, pH = 3, 4, 5, or 7) [15]. These discs were allowed to incubate for 4 h at room temperature. At the 0.5, 1, 2, 3, and 4 h, 0.5 ml of solution was removed from each well in order to check its pH and fluoride concentration (by adding 0.05 ml of total ionic strength adjustment buffer (TISAB III) to adjust the total ionic strength).

### Influence of pH changes on fluoride release of G-Is with biofilms

After 51 h-old *S. mutans* biofilms were formed on HA or G-Is discs in the slow fluoride release phase, the biofilms were transferred to a 12-well plate containing a salt solution of varying pH (pH = 3, 4, 5, or 7). These biofilms were allowed to incubate for 4 h at room temperature. At the 0.5, 1, 2, 3, and 4 h, the pH values and fluoride concentrations of the solution were determined.

### Influence of fluoride on acid production of biofilms

The influence of fluoride on the acid production of *S. mutans* biofilms was determined using a glycolytic pH drop assay [15]. The 51 h-old biofilms formed on HA discs were transferred to a salt solution (pH 7.0) containing 0, 1, 3, or 6 ppm F^−^. The pH was adjusted to 7.0 using 0.2 M KOH before glucose (final concentration: 1% w/v) was added. The subsequent decrease in pH over 4 h was assessed using a glass electrode. We determined the test fluoride concentrations based on the range of G-Is fluoride release established in a previous study [8].

### Interrelationship between fluoride release of G-Is and biofilm acid production during biofilm formation

To investigate the interrelationship between G-Is fluoride release and biofilm acid production, the pH values and fluoride concentrations of the old culture media replaced at 22, 31, 46, and 51 h during 51 h-old *S. mutans* biofilm formation on HA or G-Is discs were determined. These results were stratified by time or type of G-Is.

### Change in composition of biofilms on G-Is discs

To determine whether the composition had changed, we used microbiological and biochemical methods to analyze the dry weight, CFUs and water-insoluble EPSs of 51 h-old *S. mutans* biofilms on G-Is discs (see Microbiological and biochemical studies of biofilms in Supplementary). Confocal laser scanning microscopic (CLSM) studies as described by Jeon et al. [16] were also performed to confirm the results of the biochemical studies (see CLSM Studies of biofilms in Supplementary). The bio-volume (μm^3^/μm^2^), thickness (μm), and coverage (%) of the bacteria and EPSs were quantified using the COMSTAT image-processing software [17]. The bio-volume was defined as the volume of the biomass (μm^3^) from a certain biofilm area divided by the surface area of the certain substratum (HA discs) (μm^2^). The coverage (%), which is the fraction of the area occupied by biomass in each image of a stack, reflects how efficiently each stack is colonized by bacteria or EPSs. The three-dimensional architecture of the biofilms was visualized using Imaris 8.0.2 (Bitplane, Zurich, Switzerland).

### Statistical analyses

To determine the relationship between pH and G-Is fluoride released, power, polynomial, and linear curve fitting were performed. The determination coefficient (R^2^), which describes the percentage of the variation in one variable that can be explained by the linear relationship with another variable, of each fitted line was also calculated. All experiments were performed in duplicate, and at least three different experiments were conducted. Data are presented as mean ± standard deviation. Intergroup differences were estimated using one-way analysis of variance, followed by a post hoc multiple comparison test (Tukey test). Values were considered statistically significant when the *P* value was < 0.05.

## Results

### Influence of pH changes on fluoride release of G-Is without biofilms

**Figure 1.**
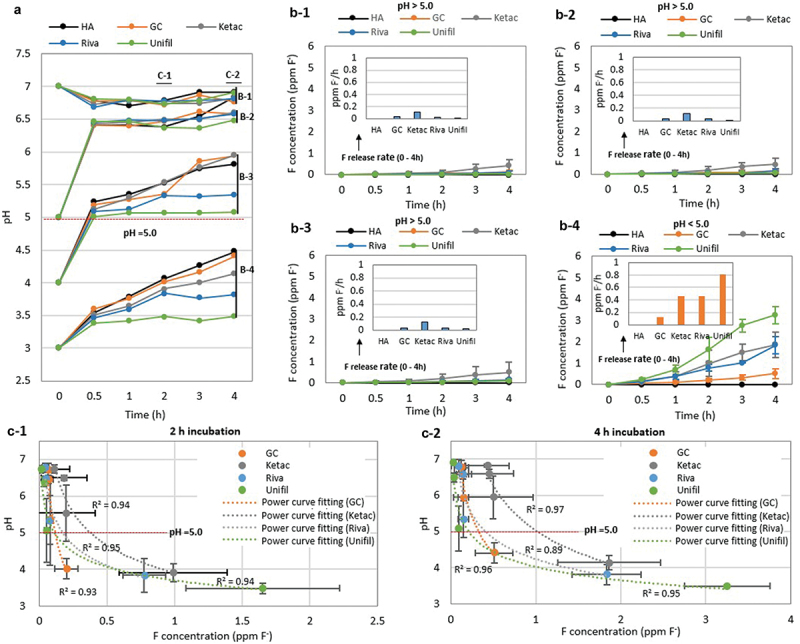
Influence of pH on fluoride release of G-Is without biofilms. (a) Changes in pH and (b) fluoride concentration during incubation of G-Is in a salt solution over time. (c) Relationships between pH and fluoride concentration. (b-1, b-2, b-3) Changes in fluoride concentration in pH higher than and (b-4) less than 5. (c-1) Relationships between pH and fluoride concentration after 2 and (c-2) 4 h incubation. Four commercially available G-Is restorative materials were selected for this study: GC Fuji Filling LC (GC), Ketac Fil Plus Aplicap (Ketac), Riva self-cure HV (Riva), and Unifil Flow (Unifil). HA refers to hydroxyapatite.

**Figure 2.**
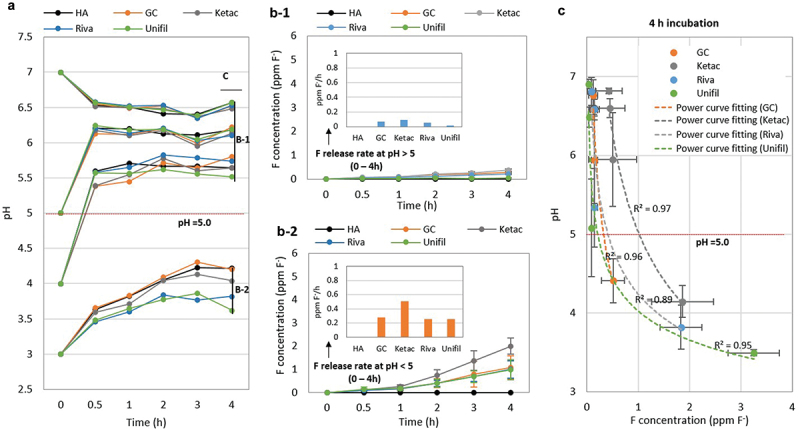
Influence of pH on fluoride release of G-Is with *S. mutans* biofilms. (a) Changes over time in pH and (b) fluoride concentration during incubation of G-Is with the biofilms in a salt solution. (c) Relationship between pH and G-Is fluoride release after 4 h incubation. Four commercially available G-Is restorative materials were selected for this study: GC Fuji Filling LC (GC), Ketac Fil Plus Aplicap (Ketac), Riva self-cure HV (Riva), and Unifil Flow (Unifil). HA refers to hydroxyapatite.

**Figure 3.**
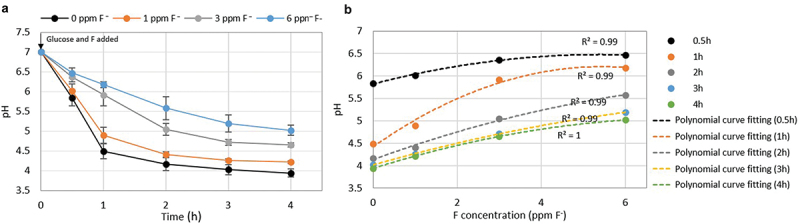
Influence of fluoride on biofilm acid production. (a) Influence of fluoride on biofilm acid production and (b) relationships between pH and fluoride over time.

**Figure 4.**
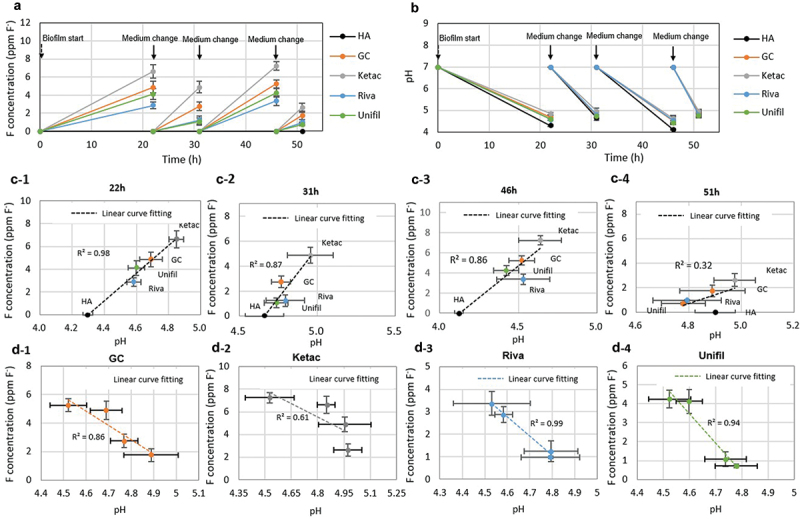
Interrelationship between fluoride release and biofilm acid production during 51h-old *S. mutans* biofilm formation on G-Is and HA discs. (a) Changes in fluoride concentration and (b) pH of old culture medium during biofilm formation. (c) Relationships between fluoride and pH according to biofilm formation time at 22, 31, 46, and 51 h (c-1, c-2, c-3, c-4) and (d) Relationships between fluoride and pH according to type of G-Is (GC, Ketac, Riva, and Unifil) (d-1, d-2, d-3, d-4). Four commercially available G-Is restorative materials were selected for this study: GC Fuji Filling LC (GC), Ketac Fil Plus Aplicap (Ketac), Riva self-cure HV (Riva), and Unifil Flow (Unifil). HA means hydroxyapatite.

The pH values of the solution in which the G-Is discs were incubated without biofilms changed with time (Figure 1(a)). After 4 h of incubation, the pH of the solution fell between 3.5 and 7. The fluoride release of G-Is in solutions with pH levels higher than 5 did not change significantly over time, but increased rapidly in solutions with pH levels lower than 5 (Figure 1(b-1–b-4)). In general, the fluoride release of G-Is without biofilms was dependent on the solution’s pH, increasing rapidly for pH < 5, regardless of incubation time, in the shape of a power curve (R^2^ = 0.89–0.97) (Figure 1(c-1, c-2)).

### Influence of pH changes on fluoride release of G-Is with biofilms

The pH values of solution changed over time even when *S. mutans* biofilms were formed on the G-Is discs (Figure 2(a)). The fluoride release of G-Is with biofilms did not change significantly over time in pH higher than 5, but increased rapidly in pH less than 5 (Figure 2(b-1, b-2)). The cumulative fluoride concentration at 2 and 4 h were 0.5–1 and 1–2 ppm F^−^ respectively for pH < 5, respectively (Figure 2(b)). The fluoride release pattern after 4 h incubation followed a power curve (R^2^ = 0.89–0.97) (Figure 2(c)), similar to our findings in Figure 1(c).

### Influence of fluoride on acid production of biofilms

In this study, fluoride in the range of 1–6 ppm F^−^ resulted in a higher solution pH by inhibiting acid production by *S. mutans* biofilms (Figure 3(a)). As shown in Figure 3(b), the fluoride concentration inhibited acid production of the biofilms, which followed a polynomial curve, regardless of the incubation time (R^2^ = 0.99–1).

### Interrelationship between fluoride release of G-Is and biofilm acid production during biofilm formation

The changes in fluoride release and pH levels during *S. mutans* biofilm formation on G-Is discs can be considered the net result of the two-way relationship between G-Is fluoride release and biofilm acid production. As shown in Figure 4(a, b), the fluoride concentration and pH values of the old culture media were dependent on the biofilm formation time and the G-Is type. Different discs released different amounts of fluoride. We found an inverse correlation between the amounts of the released fluoride and the acidity in the spent media among G-Is regardless of the biofilm formation time (Figure 4(c-1-c-4)). This relationship followed a linear curve (R^2^ = 0.32–0.98), which suggested that the materials that released greater amounts of fluoride exhibited greater suppressions of acid production by *S. mutans*. For each G-Is over different time points, there was a correlation between the acidity and the amounts of the released fluoride in the spent media (Figure (4d-1-d-4)). This relationship followed the shape of a linear curve (R^2^ = 0.61–0.99).

### Change in composition of biofilms on G-Is discs

**Figure 5.**
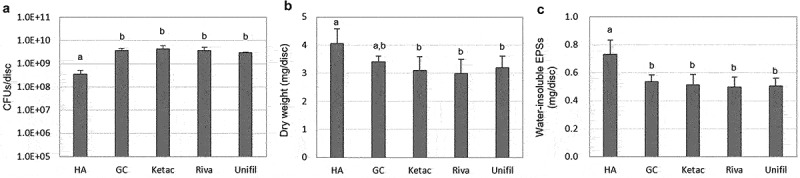
Changes in composition of the 51 h-old *S. mutans* biofilms on G-Is discs. (a) Changes in CFUs, (b) dry weight, and (c) water-insoluble EPSs. Values followed by the same superscript are not significantly different from each other (*P* > 0.05).

**Figure 6.**
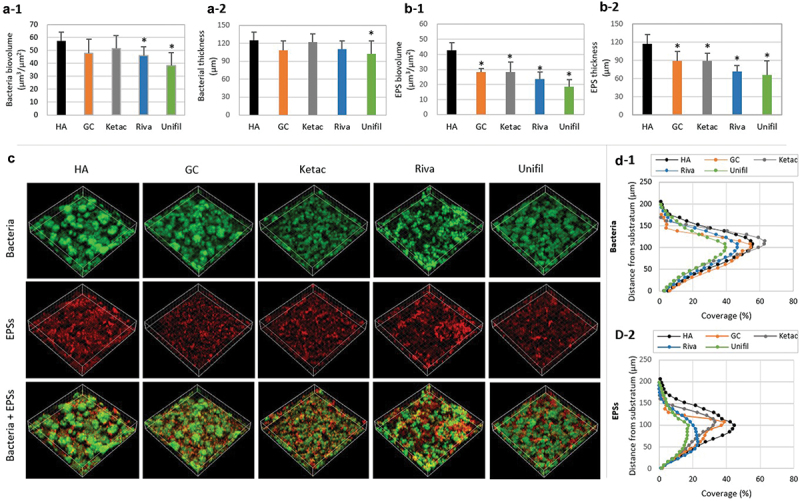
Changes in the 51-h-old *S. mutans* biofilms on G-Is or HA discs as assessed by CLSM study. (a) Changes in bacterial cells, (b) EPSs, (c) bacterial and EPS images, (d) and coverage of bacteria and EPSs (a-1, a-2) bacterial bio-volume and thickness (b-1, b-2), EPS bio-volume and thickness (d-1, d-2). Coverage of bacteria and EPSs. Four commercially available G-Is restorative materials were selected for this study: GC Fuji Filling LC (GC), Ketac Fil Plus Aplicap (Ketac), Riva self-cure HV (Riva), and Unifil Flow (Unifil). HA refers to hydroxyapatite.

**Figure 7.**
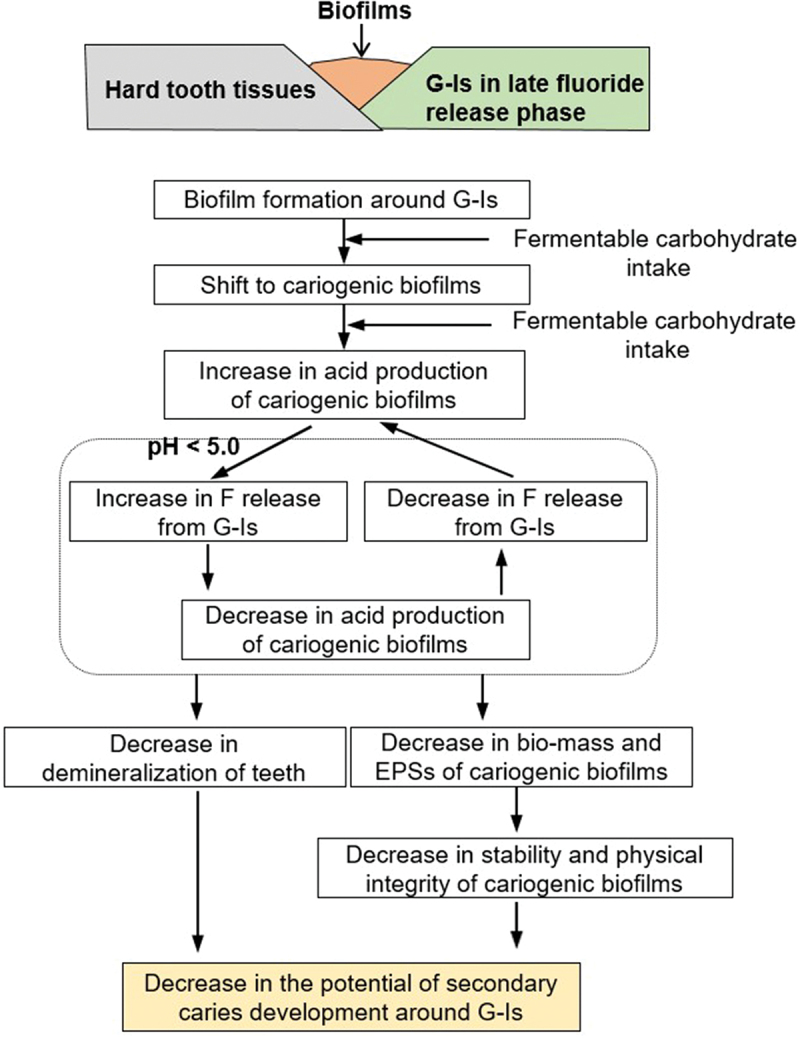
Putative mode of action of glass ionomers (G-Is) in the slow fluoride release phrase against cariogenic biofilms and subsequent secondary caries development.

Due to the interrelationship between G-Is fluoride release and biofilm acid production, the composition of the 51 h-old *S. mutans* biofilms changed. The dry weight and amount of water-insoluble EPSs of the G-Is biofilms were reduced, compared to those of the HA discs (*P* < 0.05) (Figure 5(b, c)). Conversely, the number of bacterial CFUs of these biofilms increased sharply (*P* < 0.05) (Figure 5(a)). However, CLSM analysis showed that, despite an increase in bacterial CFUs, the bio-volume, thickness, and coverage of bacteria in the G-Is biofilms were all found to be either reduced or similar to those of the HA discs (Figure 6(a-1, a-2, d-1)). The bacterial micro-colony of the biofilms on G-Is discs appeared to be smaller and more scattered than those of the HA discs (Figure 6(c)). The bio-volume, thickness, and coverage of EPSs in the G-Is biofilms were also less than those of the HA discs (*P* < 0.05) (Figure 6(b-1, b-2, d-2)). Overall, these results indicate that fluoride released from G-Is during the slow fluoride release phase may have an inhibitory effect on cariogenic biofilms. The putative stepwise mode of action of G-Is against cariogenic biofilms is described in Figure 7.

## Discussion

Multiple studies have demonstrated the therapeutic effect of fluoride releasing G-Is, particularly with regard to their ability to enhance remineralization and inhibit demineralization of the dental hard tissues [7,18]. Surprisingly, research on the inhibitory effects of fluoride releasing G-Is on cariogenic biofilms and the subsequent development of secondary caries remains poorly defined, despite the well-established causal relationship between cariogenic biofilms and dental caries. Therefore, in this study, we established a complete *in vitro* experiment model to confirm the interaction between fluoride release of G-Is and acid production of cariogenic biofilm, and further demonstrated the prospective effect of this reciprocal relationship on cariogenic biofilm virulence and secondary caries development.

In this study, fluoride release of G-Is without biofilms increased rapidly in the shape of a power curve (R^2^ = 0.89–0.97) for pH < 5 (Figure 1(c-1, c-2), Figure 2(c)), which means that at least 89% of the variability in the fluoride release can be explained by the decrease of pH. Several previous studies also reported that the pH of solutions had a dramatic effect on G-Is fluoride release, with much more fluoride ions being released at pH < 5 [13,19]. This clearly indicates that pH is a major factor influencing G-Is fluoride release. However, previous studies have not clearly described whether G-Is with biofilms exhibit a similar pattern of fluoride release as G-Is without biofilms, as described above. This result is of particular interest because pH changes associated with dental caries are mainly caused by cariogenic biofilms. In this study, we found that fluoride release from G-Is follows a power curve regardless of biofilm formation (Figures 1(c-1, c-2), Figure 2(c)). Thus, our results clearly indicate that pH plays a major role in the fluoride release of G-Is independently of biofilm formation, for pH < 5.

Decrease in pH due to acid production of cariogenic biofilms in response to dietary carbohydrates intake is a key factor in the development of dental caries. Paradoxically, decrease in pH also acted as a major factor in increasing G-Is fluoride release (Figure 2(c)). These results strongly suggest that the degree of secondary caries development around G-Is may be associated with the simultaneous interaction between cariogenic biofilm acid production and pH-dependent fluoride release. As expected, our results revealed a two-way relationship between biofilm acid production and G-Is fluoride release (Figure 4). As shown in Figure 4(c, d), lower pH levels from increased biofilm acid production resulted in increased G-Is fluoride releases, which in turn worked to suppress acid production of cariogenic biofilms. These results clearly indicate that the relationship between cariogenic biofilm acid production and G-Is fluoride release is not unidirectional but bidirectional.

It has been well documented that EPS formation of *S. mutans* is an important virulence in dental caries pathogenesis [20]. In this study, we found that G-Is reduce EPS formation (weight, bio-volume, and thickness) of *S. mutans* biofilms during biofilm formation (Figure 5(c), Figure 6(b-1, b-2)). The disc coverage of the EPSs was also significantly reduced (Figure 6(d-2)). These results may be due to the effect of fluoride that is mainly released in the pH range < 5 though these fluoride concentrations were very low; the cumulative fluoride concentration for 2 and 4 h were 0.5–1 and 1–2 ppm F^−^ respectively, for pH < 5 (Figure 2(b)). However, many previous studies have reported that even low concentrations of fluoride (1 ppm F^−^) can influence *S. mutans* EPS formation [21]. Additionally, previous studies have reported that G-Is have fluoride systems that can be recharged by exposure to fluoride in solution [22], suggesting that the use of supplementary fluoride additives after G-Is oral cavity placement can stimulate and prolong the anti-cariogenic biofilm activity.

In this study, we found the bacterial CFUs of *S. mutans* biofilms on G-Is significantly greater than those of the HA disc biofilms (Figure 5(a)). This increase may be due to the biofilms compensating for fluoride-induced suppression of bacterial bioactivity. Previous studies also yielded similar results [8]. However, the bio-volume, thickness, and coverage of bacterial cells in the G-Is biofilms were similar to or less than those of the HA discs (Figure 6(a-1, a-2, d-1)). Considering changes in CFUs and bio-volume of bacterial cells together, it is possible to assume that the shape of the bacterial micro-colonies formed on the G-Is were different from those formed on the HA discs. As shown in Figure 6(c), the bacterial micro-colony of the G-Is biofilms was smaller and more scattered than that of the HA disc biofilms. The maintenance or reduction of the G-Is biofilm thickness and coverage may be closely related to the change in the biofilm bio-volume.

All in all, data in this study and from previous investigations suggest the following mode of action of G-Is in the slow fluoride release phase against cariogenic biofilms and subsequent secondary caries development (Figure 7): (1) A shift from dental biofilms to cariogenic biofilms around G-Is by environmental disturbances such as fermentable carbohydrate intake [23]; (2) Increase in acid production (acidification) of cariogenic biofilms by fermentable carbohydrate intake (Figure 3(a)); (3) Increase in G-Is fluoride release in response to biofilm acidification, particularly in the pH range < 5 (Figure 2(c)); (4) Fluoride-dependent suppression of cariogenic biofilm acid production (Figure 3(a)), which subsequently results in a decrease in fluoride release and an increase in biofilm acid production in response to fermentable carbohydrate intake: this step demonstrates the reciprocal relationship between biofilm acid production and G-Is fluoride release; (5) Decrease in biomass and EPS formation of cariogenic biofilms due to increased fluoride release (Figure 6); (6) Decrease in stability and physical integrity of cariogenic biofilms due to decreased biofilm biomass and EPS formation; (7) Decrease in the potential of secondary caries development around G-Is.

## Conclusions

In the formation of cariogenic biofilms on/around G-Is, fluoride release from G-Is and acid production from cariogenic biofilms influence each other. Acid production by the cariogenic biofilm, particularly at < pH 5, promotes the release of G-Is fluoride. Conversely, the release of G-Is fluoride inhibits the acid production of the cariogenic biofilm. This two-way interaction results in the reduction of virulence, such as EPS formation and cariogenic biofilm bio-mass, which may reduce the potential of secondary caries development around G-Is. These results suggest that G-Is can play an important role in reducing the virulence of cariogenic biofilms and preventing subsequent formation of secondary caries if the fluoride release rate is adequate.

## Supplementary Material

Supplemental Material
