# Supplementary material for: The reciprocal interaction between fluoride release of glass ionomers and acid production of Streptococcus mutans biofilm
Source: J Oral Microbiol. 2022 Mar 23;14(1):2055267. doi: 10.1080/20002297.2022.2055267 (PMC8956307; doi:10.1080/20002297.2022.2055267)
Supplement: Supplemental Material [file ZJOM_A_2055267_SM4348.docx]

**The reciprocal interaction between fluoride release of glass ionomers and acid production of *Streptococcus mutans* biofilm**

**Supplementary information**

**Supplementary Table.** Characteristics of G-I restorative materials* used in this study

| Product name | Curing | Composition (wt.%) | Manufacturer |
| --- | --- | --- | --- |
| GC Fuji Filling LC (GC) | Light-cured | Paste A: Alumino-fluoro-silicate glass (amorphous) (75–85%); 2-hydroxyethyl methacrylate (10–12%); Urethanedimethacrylate (2–5%).  Paste B: Distilled water (20–30%); polyacrylic acid (20–30%); urethanedimethacrylate (12–15%); silicone dioxide (fumed/amorphous) (10–15%). | GC corporation, Japan |
| Ketac^TM^ Fil Plus Aplicap^TM^ (Ketac) | Light-cured | Glass powder (>99%).  Liquid: copolymer of acrylic acid-maleic acid (35–55%), water (40–55%), tartaric acid (5–10%). | 3 M ESPE, Dental Products, Germany |
| Riva self-cure HV (Riva) | Light-cured | Polyacrylic acid (20–30%).  Tartaric acid (10–15%).  Fluoroaluminosilicate glass (90–95%).  Polyacrylic acid (5–10%). | SDI Limited, Australia |
| UniFil Flow (Unifil) | Light-cured | Polyacrylic acid (20–30%).  Tartaric acid (10–15%).  Fluoroaluminosilicate glass (90–95%).  Polyacrylic acid (5–10%). | GC Corporation,  Japan |

* The G-I discs were manufactured using polytetrafluoroethylene (Teflon) molds with a metal holder and glass slides to cover each face. GC and Unifil specimens were light-cured for 40 s on each face using a light curing unit (G-Light, GC Corp., Japan). After curing, all specimens were polished sequentially with # 800- #1200 sand papers. Then, the specimens were placed in a desiccator at room temperature.


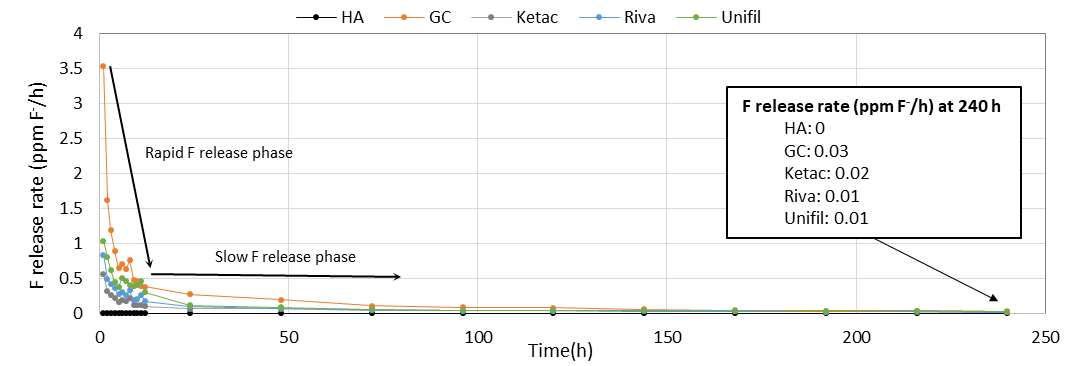


**Supplementary Figure 1.** Fluoride release of G-I discs* that immersed in 50 mM potassium phosphate (PP) buffer for 240h.

* To evaluate fluoride release from G-I discs, the discs that were prepared and kept in a desiccator were immersed in individual 24-well plates containing 50 mM potassium phosphate (PP) buffer at pH 7.0 (2.8 ml/well). After 1 h immersion at room temperature, each disc was transferred to new PP buffer, which was repeated every hour for 12 h. At 24 h, the PP buffer was changed to new one. From 24 h to 240 h, the buffer change was repeated every 24 h. The fluoride concentration in the PP buffer was measured after adding 280 ml of total ionic strength adjustment buffer (TISAB III) to 2.8 ml of old buffer. To calculate the fluoride release rate, the fluoride concentration in the old buffer was divided by the immersion time.

* Base on fluoride release rate, the G-I discs that immersed in 50 mM potassium phosphate (PP) buffer for 240 h were defined as G-Is in the slow fluoride release phase and used for this study.


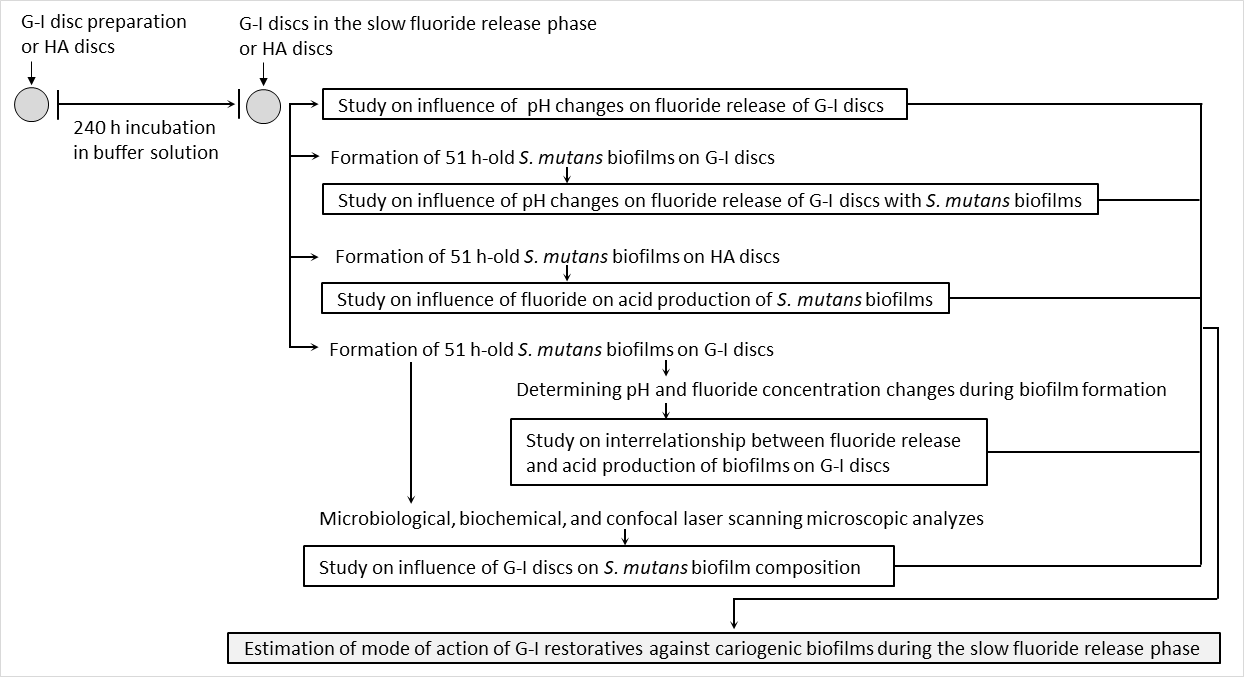


**Supplementary Figure 2.** Experimental scheme for this study.

**Microbiological and Biochemical Studies of Biofilms**

The dry weight, CFUs, and level of water-insoluble EPSs were determined as described elsewhere [1,2]. Briefly, the 51 h-old biofilms were transferred into 2 ml of 0.89% NaCl and sonicated in an ultrasonic bath (Power sonic 410; Hwashin Technology Co., Seoul, Korea) to disperse the biofilms. The suspension was homogenized by sonication (VCX 130PB; Sonics and Materials Inc., Newtown, CT, USA) for 30 s after adding 3 ml of 0.89% NaCl. An aliquot (100 μl) of the homogenized suspension was serially diluted and plated to determine the number of CFUs. To determine dry weight and amount of water-insoluble EPSs, the remaining solution (4.9 ml) was centrifuged (3000 g) for 20 min at 4°C. The biofilm pellet was then lyophilized and weighed to determine the dry weight. The water-insoluble EPSs were extracted from the dry pellet using 1 N sodium hydroxide before determination of the polysaccharide amount using a phenol-sulfuric acid assay.

**CLSM Studies of Biofilms**

At the end of experimental period (51 h-old biofilms), the change in biofilm formation on G-I discs was investigated by simultaneously in situ labeling of EPSs and bacterial cells as described by Jeon et al. [3]. Briefly, 1 µM of Alexa Fluor® 647-labeled dextran conjugate (10,000 MW; absorbance/fluorescence emission maxima 647/668 nm; Molecular Probes Inc., Eugene, OR, USA) was added to the culture medium at 0, 22, 31 and 46 h. The ﬂuorescence-labelled dextran serves as a GTF primer and can be incorporated into newly formed EPS during synthesis of the EPS matrix. After 51 h, the bacterial cells in the biofilms were labeled by incubation with 2.5 µM SYTO 9 green fluorescent nucleic acid stain (480/500 nm; Molecular Probes Inc.) for 30 min. CLSM imaging of the biofilms was performed using a LSM 510 META (Carl Zeiss, Jena, Germany) microscope equipped with argon ion and helium-neon lasers.

**References**

[1] Koo H, Schobel B, Scott-Anne K, Watson G, Bowen WH, Cury JA, Rosalen PA, Park YK. Apigenin and tt-farnesol with fluoride effects on *S. mutans* biofilms and dental caries. J Dent Res. 2005; 84(11):1016–1020.

[2] Cai JN, Choi HM, Jeon JG. Relationship between sucrose concentration and bacteria proportion in a multispecies biofilm. J Oral Microbiol. 2021; 13(1):1910443.

[3] Jeon JG, Klein MI, Xiao J.et al. Influences of naturally occurring agents in combination with fluoride on gene expression and structural organization of *Streptococcus mutans* in biofilms. BMC Microbiology. 2009; 9:228.
